# A Comprehensive Review on Characterization of Pepper Seeds: Unveiling Potential Value and Sustainable Agrifood Applications

**DOI:** 10.3390/foods14111969

**Published:** 2025-06-01

**Authors:** Alicia Dobón-Suárez, Pedro Javier Zapata, María Emma García-Pastor

**Affiliations:** 1 Department of Agri-Food Technology, Institute for Agri-Food and Agro-Environmental Research and Innovation (CIAGRO), University Miguel Hernández, Ctra. Beniel km. 3.2, Orihuela, 03312 Alicante, Spain; adobon@umh.es; 2 Department of Applied Biology, Institute for Agri-Food and Agro-Environmental Research and Innovation (CIAGRO), University Miguel Hernández, Ctra. Beniel km. 3.2, Orihuela, 03312 Alicante, Spain

**Keywords:** byproduct valorisation, *Capsicum annuum* L., functional ingredient, nutritional value, pepper seeds, sustainable food development

## Abstract

Pepper (*Capsicum annuum* L.) processing generates significant byproducts, with seeds emerging as a promising resource due to their rich content of oils, proteins, phenolic compounds and minerals. This comprehensive review critically evaluates the existing literature on the characterization of pepper seeds, highlighting their significant nutritional value and diverse bioactive constituents. The substantial oil content, characterized by a high proportion of unsaturated fatty acids, such as linoleic and oleic acids, positions pepper seeds as a valuable source for edible oil and potential biofuel production. In addition, the presence of significant amounts of proteins, carbohydrates, dietary fibre and essential amino acids underlines their potential for the development of functional foods and dietary supplements. The current review also summarizes the findings on the phenolic profile and antioxidant activities of pepper seeds, indicating their relevance for nutraceutical and cosmetic applications. Finally, the potential utilization of pepper seeds in various agri-food industrial applications, such as food condiments, biostimulants, and biomass for energy, is discussed, promoting sustainability and a circular bioeconomy approach to valorise this underutilized resource. This systematic review summarizes current knowledge, identifies knowledge gaps, and highlights the potential of pepper seeds as a sustainable and economically viable alternative in multiple sectors.

## 1. Introduction

Peppers (*Capsicum annuum* L.) are considered to be one of the most widely consumed vegetable crops on the global stage, cultivated in substantial quantities across diverse regions of the world [1,2]. The primary producers of both sweet and hot pepper fruit are China, Mexico, Indonesia, Turkey, Spain, and the USA. For instance, the annual Spanish production of sweet and chilli peppers was up to 1416 million tons in 2023 [3]. Peppers are classified into three primary categories based on their colour—red, green, or yellow—and are used as a vegetable or a spice, whether fresh or dried [4]. Furthermore, they undergo various processes, including dehydration, pickling, freezing after slicing or dicing, and transformation into final products, such as sauce, puree, flakes, powder, and oleoresin [5]. Processing techniques significantly alter the biochemical composition of pepper and its byproducts, influencing the concentration and bioaccessibility of valuable compounds, such as capsaicinoids, phenolics, and carotenoids [5].

The consumption of fresh peppers has been shown to provide significant health benefits due to their high nutritional value and antioxidant content [6]. They are a source of vitamin C, provitamin A, E, carotenoids, chlorophylls, phenolic acids, and flavonoids [7,8]. Peppers represent an exceptional source of antioxidants, whose antioxidant profile plays a key role in reducing oxidative stress. In this sense, studies have indicated that antioxidant compounds are effective in scavenging reactive oxygen species (ROS) and protecting cellular structures against free-radical damage caused by oxidative processes [9]. Scientific literature supports the correlation between free radicals and the development of various pathologies, including diabetes mellitus, cardiovascular disease, cancer and neurodegenerative diseases [10,11]. However, bioactive compounds present in *Capsicum annuum* species have been shown to reduce the production of free radicals and possess anti-inflammatory properties, stimulate the immune system and reduce the likelihood of developing chronic disorders and diseases [12,13,14].

The substantial antioxidant content of peppers represents a significant opportunity for the valorisation of byproducts within the agri-food sector. Residues generated during the processing of peppers, including peels, seeds, and pulp remnants, have been found to be abundant in bioactive compounds such as phenolics, carotenoids, vitamins, minerals, and dietary [15,16,17]. These byproducts have the potential to be repurposed for the extraction of natural antioxidants, food colourants, and nutritional supplements, thereby fostering sustainability and mitigating waste [18]. In addition, the fibre present in these residues can be incorporated into the formulation of functional foods, enhancing their nutritional value and promoting digestive health [19]. Consequently, there is a necessity for research into extraction and processing technologies for pepper byproducts, with the objective being to maximize the utilization of these resources and to develop innovative products that confer benefits to both human health and the environment.

## 2. Literature Search and Selection Criteria

A literature search was conducted by querying the Scopus database using the following search string: (‘*Capsicum annuum* L.’) AND (‘pepper seeds’) AND (‘byproduct valorisation’) AND (‘functional ingredient’) AND (‘nutritional value’) AND (‘sustainable applications’) AND (‘circular bioeconomy’), limiting the time span to the past 5 years. This comprehensive review critically evaluates the existing literature on the characterisation of pepper seeds, highlighting their significant nutritional value and diverse bioactive constituents, as well as their potential applications in the agri-food and non-food industries. The screening process yielded approximately 200 publications, which were evaluated based on their abstracts. The inclusion criteria were as follows: works written in English with an available abstract; works that clearly referenced the nutritional value of pepper seeds; and works focused on the following types of application: condiment use, plant biostimulant use, nutraceutical use, cosmetic use and biofuel use. The subsequent paragraphs describe in detail the characterisation of pepper seeds, unveiling their potential value and sustainable applications in the aforementioned agri-food and non-food industry categories, based on an analysis of the recent literature. Nevertheless, the main knowledge gap, as far as we are aware, is the lack of literature on the revalorisation of pepper seeds. For example, Sanatombi [5] reviewed the sustainable strategies for valorisation of pepper waste and their potential applications, but the study lacks focus on pepper seeds because it takes a general approach to all crop plant waste (e.g., stalks, placenta, peels, seeds, and other processing byproducts). Therefore, five publications were selected to demonstrate the various approaches on which this review is based, as shown in Table 1, to highlight the potential value of these seeds in the agri-food and non-food industries.

## 3. Addressing Pepper Waste: A Focus on the Revalorisation of Seeds

In recent years, the substantial amount of waste generated during pepper production and processing, including seeds, has posed a considerable environmental challenge. This is because these byproducts are often sent to landfill, contributing to pollution [5]. In this context, modern industrial and agricultural sustainability strategies emphasise the sustainable utilisation of these byproducts to generate value, with the aim of minimising losses and waste from peppers. These objectives are crucial for mitigating the environmental and economic consequences of food waste. Despite their status as a byproduct, pepper seeds are rich sources of beneficial compounds such as proteins, dietary fibre, fats, minerals, amino acids, vitamins, capsaicinoids, phenolics, flavonoids and carotenoids, which are recognised for their health-promoting properties, such as antioxidant activity [19,20]. Given these properties, modern industrial and agricultural sustainability strategies emphasise the sustainable utilisation of these byproducts, with the dual objectives of generating value and minimising losses and waste.

This approach has been demonstrated to optimize available resources whilst concomitantly fostering the development of new food products, thus contributing to environmental sustainability and economic growth [21,22]. The reduction of food loss and waste has been demonstrated to engender substantial environmental benefits, whilst concomitantly enhancing global food security, a pivotal element in the overarching pursuit of sustainable development. In response to these challenges, the industry has intensified research into more environmentally friendly methods of food and nutritional supplement production [23].

**Figure 1:**
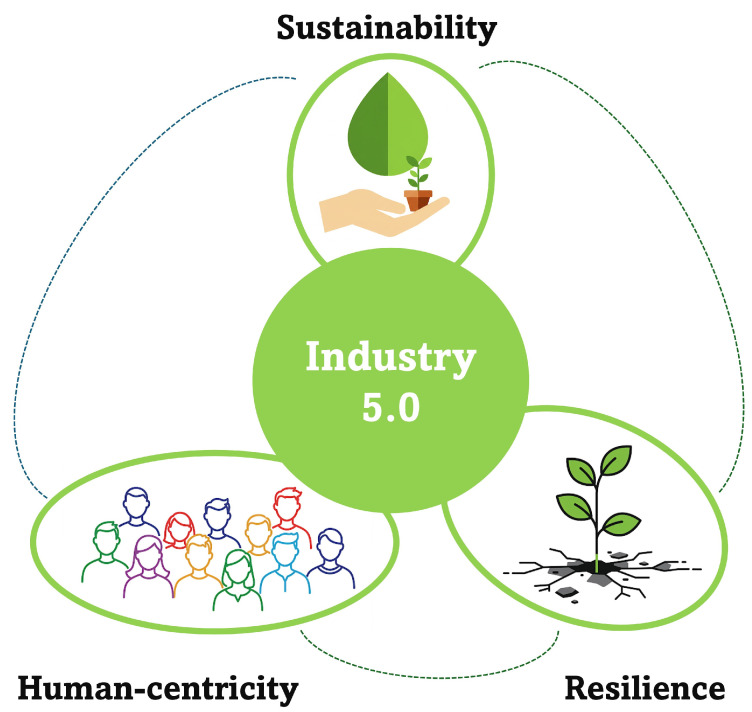
Core principles of the framework of food industry 5.0: Sustainability, human-centricity and resilience. The dashed line establishes a connection among them.

A particularly encouraging approach involves the revalorisation of food waste, a process which entails the extraction of bioactive compounds for the purpose of creating functional foods and nutraceuticals. This strategy is expected to yield a number of significant benefits, including both economic and health-related advantages [24]. Transforming pepper seeds into high-value functional ingredients is a practical ‘zero-waste’ approach that promotes the development of profitable industries simultaneously [20]. This strategy is vital for reducing the environmental burden and is essential for transitioning to sustainable food systems and fostering a circular bioeconomy [25]. There are many sustainable applications for valorised pepper seeds, including the extraction of bioactive compounds for use in incorporating functional foods, nutraceuticals and pharmaceuticals into food products to enhance nutritional and functional attributes [20,26,27,28]. Additionally, valorised pepper seeds can be used for conversion into materials such as biochar and activated carbon for environmental applications or energy storage [29,30]. The successful implementation of these sustainability objectives depends on developing efficient, economically feasible ‘green’ extraction technologies with minimal environmental impact, and on carrying out stringent safety evaluations to ensure the absence of contaminants. Ultimately, the sustainable valorisation of pepper seeds through these various applications is a critical step towards waste reduction and realising a more circular bioeconomy. This approach is consistent with the tenets of the circular economy, an emerging economic model that advocates the reuse of organic waste as raw materials, thereby reducing waste generation, optimizing resource efficiency and improving the safety and security of global food supply [31]. In accordance with the three core elements of the framework of food industry 5.0 (Figure 1), the food industry is adopting a sustainable approach and the principles of the circular economy with a view to reducing food waste from farm to fork [32]. A future in which technological advancement serves as a catalyst for positive social and economic impacts, thereby aligning industrial progress with the well-being and active participation of humanity, is conceptualised by this approach.

The traditional linear economic model, known as the ‘make–use–dispose’ paradigm, has been superseded by a circular economy model that is based on the ‘5Rs’—reduce, recycle, reuse, recover, and restore—creating a more sustainable and regenerative system [33]. The agro-industrial sector has undergone significant transformation due to modernization and industrialization, leading to an increase in agri-food waste [34]. The organic waste from fruits and vegetables has been identified as a significant source of bioactive compounds, including carbohydrates, dietary fibre, proteins, lipids, organic acids, minerals, polyphenols, and phytosterols [35]. These valuable components open new possibilities for high-value applications, such as the production of biofuels, animal feed, and food supplements. Post-harvest losses in peppers present a substantial challenge to the achievement of the sustainable development goals (SDGs). These losses have a direct impact on SDG 2 (Zero Hunger) by reducing the availability of nutritious food, and on SDG 12 (Responsible Consumption and Production) by contributing to food waste along the supply chain. Furthermore, the economic repercussions of these losses have a profound effect on SDG 1 (Poverty Eradication) for farmers and associated stakeholders, particularly in developing economies where post-harvest infrastructure may be deficient [36].

Numerous studies have demonstrated that pepper losses can be significantly attributed to several factors, including inadequate storage conditions, pathogen infections and mechanical damage [37,38]. In addition, external factors occurring outside the food supply chain have been shown to induce substantial levels of post-harvest losses. These factors can be categorized into two primary classifications: environmental factors and socio-economic patterns or trends [39]. Consequently, there is an imperative for the implementation of improved post-harvest handling and storage technologies to minimize such losses and advance the SDGs related to food security and sustainable development [40]. Sweet peppers, for instance, experience approximately 40% post-harvest loss each year due to their perishable nature, limited shelf-life, and susceptibility to many diseases [41]. The most effective method currently used to extend the shelf-life of fresh peppers and prevent spoilage is to apply low temperatures [42,43].

Post-harvest rot significantly affects fleshy vegetables such as bell peppers, primarily due to fungal and bacterial infections. Fungi are particularly problematic due to sporulation, rapid spread, adaptation to low temperatures and dormancy [44]. *Alternaria alternata* has been identified as the fungal pathogen responsible for severe fruit rotting in green bell peppers [44]. This pathogen is well known for affecting horticultural crops, particularly solanaceous species such as sweet peppers, tomatoes, potatoes, and aubergines, and can cause a variety of diseases [45,46,47,48]. Infection often occurs during storage and marketing, with the opportunistic pathogen entering through wounds, natural openings or direct cuticle penetration [49]. *Alternaria alternata* can penetrate unripe tissue, remain dormant, and resume proliferation when the fruit ripens. This process is influenced by factors such as cell wall degradation, reduced levels of antifungal compounds, the decline of proteins, changes in pH, and nutrient availability [50]. For instance, Balamurugan and Kumar [44] confirmed that *Alternaria alternata* Cap_Aa remains quiescent for ten days in inoculated green bell peppers, leading to fruit rot upon maturation. They also demonstrated its pathogenicity on tomato and aubergine, causing rot within eight to twelve days post-inoculation. Infected bell peppers showed symptoms of internal rot, including seed decay [44]. Post-harvest fruit rot in market-sourced green bell peppers may result from temperature fluctuations and/or mechanical damage. Defective or damaged fruits contribute to pepper waste.

On the other hand, the cultivation and processing of pepper fruit for minimally processed plant-based food products generate a significant amount of waste, estimated at 5–30%. This includes fruit waste (e.g., seeds, peduncle, peel, placenta, and unused fruits) as well as plant waste (e.g., stems and leaves). The potential for valorisation of these byproducts is increasingly recognized, due to their bioactive richness, and the presence of valuable compounds such as carotenoids, phenolics, dietary fibre, essential fatty acids, and vitamins [5,51]. Pepper seeds have attracted significant attention due to their substantial oil content, which is characterized by a high proportion of unsaturated fatty acids, such as linoleic and oleic acids. In addition to this, they contain significant amounts of phenolic compounds, proteins, and minerals that offer health benefits [5,52,53,54,55]. Recent studies have indicated that pepper seeds could be repurposed into valuable products such as edible oil, carbohydrate-rich flour, and protein-based ingredients [4,21,56,57,58]. Notwithstanding their nutritional and functional potential, pepper seeds constitute approximately 45% of the dry weight of peppers and are frequently discarded or utilized for low-value applications such as animal feed [59]. This represents a significant opportunity for optimizing resources and promoting sustainability within the agri-food sector. The development of innovative applications for pepper seed-derived products has the potential to reduce food waste, while simultaneously unlocking new economic and nutritional benefits.

A preliminary evaluation revealed a paucity of exhaustive scientific review literature specifically addressing pepper seeds as byproducts, and particularly with regard to their sustainable agrifood applications in the industry. Therefore, A number of authors have conducted reviews of the nutritional components and bioactivities of pepper seeds, as well as the details of their extractions from pepper seeds, further highlighting the relative scarcity of review literature dedicated to their derived byproducts [5]. Consequently, there is a necessity for dedicated scientific reviews that consolidate and analyse the potential applications and valorisation strategies for specific pepper seed byproducts, within the food and other relevant sectors.

## 4. Potential Value of Pepper Seeds: Nutritional Profile and Phytochemical Compounds

The increase in the production of agri-food byproducts has stimulated research into the valorisation of resources that are traditionally considered waste, but which have considerable nutritional and economic potential. In this context, pepper seeds emerge as a promising byproduct due to their rich composition of macronutrients, minerals and amino acids. These components not only provide high energy and functionality but also suggest applications in the development of functional foods, nutraceuticals and other innovative products [16,60,61]. This review examines the nutritional and phytochemical composition of pepper seeds, analyses the variability derived from the cultivar, and highlights its relevance for improving the sustainability and diversification of the agri-food industry.

### 4.1. Nutritional Composition of Pepper Seeds: Carbohydrates, Dietary Fibre, Proteins, Fats, Moisture and Ash

Pepper seeds show a considerable variability in their constituents, reflecting both the diversity of varieties and growing conditions, and the different processing and analytical methods used in the studies. In terms of macronutrients, the range of carbohydrates is from 43.60 to 80.89 g 100 g^−1^ [17,55,62], suggesting that these seeds can be an important source of energy (Table 2). Chouaibi et al. [55] found the lowest carbohydrate content in pepper seeds (43.60%). In the carbohydrate content of pepper seeds, the most dominant component is dietary fibre, with significant levels ranging from 26% [4] to 61% [56] (Table 2). The ratio of insoluble and soluble dietary fibre was around 10:1, according to Azabou et al. [56]. The high content of insoluble fibre in pepper seeds could represent a new ingredient in the food industry, such as pepper seed flour, which could be used to enrich various products (e.g., jams, sauces or soups) by increasing the levels of indigestible insoluble compounds [16].

Pepper seed proteins have not been extensively studied, as evidenced by the literature. As demonstrated in Table 2, pepper seeds exhibit high concentrations of crude protein (6.30–28.30 g 100 g^−1^), with the highest levels recorded by Chouaibi et al. [55]. It is acknowledged that variations in protein yield can be attributed to several factors, including cultivar, cultivation methods, climatic conditions, the ripening stage of the seeds, and the timing of the harvest. For instance, the protein content of pepper seeds from Croatian cultivars was found to be 16.5% for the Slavonka cultivar and 16.7% for the Podravka cultivar [21]. The quality of the protein is contingent upon its amino acid composition. For example, the study of Embaby and Mokhtar [62] revealed that the proteins from sweet pepper seeds, lanta seeds and nabak seed kernels had a low biological value. Consequently, these types of proteins are classified as incomplete proteins so that, in turn, these seeds necessitate supplementation with complementary proteins if they are to be utilized as a food source [63]. Pepper seeds contain most of the essential amino acids, as discussed below, and can be used as a good protein source in a variety of food applications [60,64]. Pepper seed flour, which is characterized by its elevated lysine content, has the potential to serve as a functional ingredient, with the capacity to enhance the protein quality of wheat flour, which is known to be deficient in lysine [5]. These findings suggest the possibility of using pepper seed protein as an alternative, inexpensive source of protein [5], though the dose will determine whether it will dominate the taste of food products. Hence, pepper seed flour has the potential to be used in some bakery products, as a meat replacer and as a thickening agent in soups [52]. Red pepper placenta and defatted seeds rich in protein and fibre have also been used to fortify pasta, improving the amino acid composition and total dietary fibre content of said pasta [65].

The presence of crude fat, ranging from 11.00 to 23.65 g 100 g^−1^ [17,55,56,62,63,64] is notable (Table 2), as it provides nutritionally significant lipids and essential fatty acids. The fat content of the pepper placenta was recently determined to be 3.15% dry weight (DW), and 10.40% in the defatted pepper seeds [65]. Prior to the defatting process, the seeds exhibited a fat content of 26.01% DW [65], which is congruent with the findings reported by Cvetković et al. [16]. Finally, 100 g of pepper seeds contain from 4.48 to 5.96 g of moisture [62,63] and ≈ 4.94 g ash [63] (Table 2). However, the total ash content has been reported to vary from 1.81 to 12.54 g per 100 g of seeds [17,62,63,65] (Table 2), suggesting that it is indicative of both the stability of storage and the total mineral content of these byproducts.

Additionally, bell peppers contain some nutritionally important compounds, such as vitamins (A, C, E, K, B3 and B6) and minerals (sodium, potassium, phosphorus, magnesium, calcium, iron, zinc, manganese and copper) (Table 2). In this sense, the frequent consumption of bell peppers provides essential nutrients for human health [66,67,68]. For example, fresh bell pepper consumption (100 g) provides the total recommended daily intake of ascorbic acid [69]. However, the nutritional content of bell peppers depends directly on the colour of the fruit, cultivar, growing conditions, and post-harvest processing, among other factors [66]. Therefore, vitamin C content ranged from 93 mg 100 g^−1^ FW for the creasing pepper type (green; *Capsicum annuum* L.) to 393 mg 100 g^−1^ FW for the long-point pepper type (red; *Capsicum annuum* L.), depicting a 4-fold variation between cultivars [69]. In addition, Zhuang et al. [69] found that the fully red mature long-point pepper type (red; *Capsicum annuum* L.) had significantly more vitamin C than fully developed long-point pepper fruit (green; *Capsicum annuum* L.). In this sense, the total vitamin C content, as the sum of both forms of ascorbic acid (AA) and dehydroascorbic acid (DHA), was also significantly influenced by the phenological stage of the green pepper fruit, and a 3-fold increase in total content was observed between the first and last stages (S1–S12) [70]. Thus, pepper waste and byproducts are rich sources of nutrients and bioactive compounds, such as among other things, vitamins, which exhibit anti-inflammatory, antidiabetic, antimicrobial, and immunomodulatory effects, among others [5,66]. It is evident that peppers contain a high concentration of bioactive phytocompounds. Consequently, pepper fruit waste and its processing byproducts may provide a cost-effective source for the extraction of bioactive molecules. Similar to fruits, pepper fruit waste contains several bioactive compounds such as capsaicin, dihydrocapsaicin, vitamins, carotenoids, flavonoids, and phenolic compounds (19,56,62,63,65). The other pepper plant waste, including seeds, stems, and leaves, also contains many bioactive phytocompounds [5].

Mineral elements are recognised as essential components that are vital for maintaining biological functions, including osmotic pressure, acid–base balance, blood composition, and bone structure. Although factors such as cultivar, growth environment and processing methods can influence mineral composition, pepper seeds have the potential to provide nutrients of high biological value. Potential sources of this variation include brining processes or distinct environmental conditions [17,56,62,63,65]. Macrominerals such as phosphorus, magnesium, and calcium are present, as can be seen in Table 2. These macrominerals play pivotal roles in vital processes such as metabolic regulation, energy production, and bone health. Additionally, trace minerals such as iron, zinc, manganese, and copper are present in lower concentrations (see Table 2), yet are essential for various enzymatic functions and a wide array of biochemical processes that are critical for health, growth, and overall well-being. The mineral profile of pepper seeds, as detailed in Table 2, indicates their potential as a source of these essential nutrients.

In Table 2, the amino acid profile analysis reveals an interesting nutritional capacity, with the presence of essential amino acids, such as leucine, cysteine, histidine, lysine, phenylalanine, threonine, isoleucine, tyrosine, methionine, and valine [17,62,63], whose presence varies significantly according to the cultivar of pepper seeds, suggesting that some types of seed could offer a better protein quality than others. Leucine had the highest content among the essential amino acids, ranging from 830 mg 100 g^−1^ to 4006 mg 100 g^−1^ [17,62,63], as shown in Table 2. Seven kinds of non-essential amino acids were identified, namely, glutamic acid, aspartic acid, arginine, serine, glycine, alanine, and proline [17,62,63]. In Table 2, we find that glutamic acid had the highest content among the non-essential amino acids in the pepper seeds, and it ranged from 1188 mg 100 g^−1^ to 3668 mg 100 g^−1^ [17,62,63]. In the domain of food nutrition, essential amino acids have historically been a subject of interest. These amino acids are of particular significance for humans, as the body is incapable of synthesizing them. The present review demonstrates that pepper seeds are rich in amino acids, particularly the ten essential amino acids necessary for the human body. Conversely, the seven non-essential amino acids have been shown to enhance sensory attributes, including taste and texture, in various food products, rendering them components of considerable interest to the food industry.

The extant literature suggests that pepper seeds have the potential to be a valuable byproduct within the agri-food chain. This is due to their composition, which is rich in macronutrients, vitamins, minerals and both essential and non-essential amino acids. It is proposed that, through the implementation of suitable processing methodologies, these seeds could be integrated into the development of functional foods, nutritional supplements and other innovative products. This approach would contribute to the reduction of waste and the enhancement of value in the agri-food industry, thereby promoting sustainability and the diversification of nutritional sources. Nevertheless, the nutritional composition of pepper seeds can vary significantly. This variability is primarily linked to the plant’s genetic background, cultivar and environmental conditions during growth, as well as production and processing factors [16,17]. The chemical composition of the plant is influenced by climate, soil and plant factors [71]. Even when grown under identical conditions, different plant species may respond differently to nutrient uptake from the soil [72].

A scientific review of the existing literature reveals inconsistencies in analytical methodologies employed for determining the nutritional composition of pepper seeds. Carbohydrate content is predominantly estimated by calculation of difference [17,55,62], an indirect method lacking specificity for individual carbohydrate fractions. Fibre analysis varies between crude fibre determination, which underestimates total dietary fibre [4], and potentially more comprehensive methods where specific procedures are not detailed [21]. Protein content is typically assessed via total nitrogen determination using Kjeldahl or Dumas techniques with a standard N x 6.25 conversion factor [17,21], potentially overestimating true protein due to non-protein nitrogen. Fat content is determined by solvent extraction [17,56], with variations in solvents and unspecified AOAC methods limiting reproducibility. Fatty acid composition analysis also shows divergence in transesterification and gas chromatography parameters [17,56,62]. Mineral analysis employs AOAC/AACC methods and ICP-OES [17,65] with varying levels of detail, while vitamin analysis, exemplified by HPLC-UV for B vitamins [66,68], presents a limited scope. Future research should prioritize direct, standardized analytical techniques with explicit citation of validated methods to enhance the accuracy and comparability of nutritional data for pepper seeds.

### 4.2. Functional Composition of Pepper Seeds: Total Phenolics and Flavonoids

Phenolic compounds are a vital category of secondary metabolites in plants. Peppers are considered to be a valuable source of bioactive compounds, including phenolic compounds, carotenoids, ascorbic acid (vitamin C), tocopherols (vitamin E), and capsaicinoids [73]. Analyses employing high-performance liquid chromatography coupled with tandem mass spectrometry and electrospray ionization (HPLC-ITMS) techniques have facilitated the identification of a number of polyphenolic compounds in *Capsicum annuum* fruits, including caffeic acid, coumaric acid, coumaroylquinic acid, 3-*O*-caffeoylquinic acid, ferulic acid, sinapic acid, apigenin-*O*-hexoside, and quercetin-*O*-rhamnosyl-*O*-hexoside [74]. Recent studies have highlighted the high quality of pepper seed byproduct, indicating its potential to direct the food industry towards the creation of new opportunities within the context of a circular bioeconomy. It is imperative to acknowledge the significance of recognizing food losses and waste as a potential source of biologically active components. These include antioxidants, complex soluble polysaccharides, vitamins, enzymes and fatty acids, among other bio-reagents that can be employed in a range of industries, such as the food, health, medicine and pharmaceutical industries [75].

The extant literature addresses research on various species of pepper seeds. Nevertheless, as indicated in Table 3, there is a paucity of available data regarding total phenolic and flavonoid contents in pepper seeds. The growing popularity of natural antioxidants can be attributed to the toxic and anticarcinogenic properties of synthetic antioxidants [75,76]. Consequently, the potential of natural antioxidants, such as byproducts from pepper processing, is being investigated, as are the phytochemical polyphenols, which are present in such plant materials. For instance, the total phenolic content of raw and scalded industrial jalapeño pepper seeds (*Capsicum annuum* L.) was found to have a range of values from 10.01 mg GAE g^−1^ to 13.09 mg GAE g^−1^ in dry weight (DW) base [15]. In that article, the major phenolic compounds, rutin, epicatechin and catechin, comprised 90% of the total compounds detected by HPLC of each jalapeño pepper byproduct [15]. Other studies have shown that *Podravka* pepper seeds contain 1.58 mg GAE g^−1^ DW of polyphenols, while *Slavonka* pepper seeds contain lower quantities (1.50 mg GAE g^−1^ DW) [21]. This can be explained by the fact that polyphenol content depends on the pepper cultivar.

Red pepper seed extracts exhibit a polyphenol content of 21.50 mg GAE g^−1^ DW [56], while Sim and Sil [77] have reported a content of 29.10 mg GAE g^−1^ DW in red pepper seeds. In addition, Sim and Sil [77] quantified the total flavonoid content of red pepper seed extracts, finding it to have a catechin equivalent (CAE) value of 21.27 mg g^−1^ DW. The crude extracts of red chilli (*Capsicum frutescens* L.) seeds had total phenolic content and total flavonoid content in the ranges of 7.95–26.15 mg GAE g^−1^ and 4.64–12.84 mg of rutin equivalents (RU) g^−1^ DW of extract, respectively [78]. It is evident that hot peppers exhibit a divergent polyphenol content in comparison with sweet peppers. On the other hand, the extraction with 100 mL of ethanol: acetone (50:50, *v*/*v*) of phenolics form seeds showed an extracted yield of 22.30 mg 100 g^−1^ DW and 88.60 mg 100 g^−1^ DW in seeds from red and green pepper fruit (*Capsicum annuum* L.), respectively [79]. In addition, Ahmad et al. [79] identified the phenolic profile and quantified the content of gallic acid (GA), scopoletin (SC), rosmarinic acid (RA) and resveratrol (RV), and they concluded that the seeds for the green pepper fruits exhibited more phenolic amounts, as can be observed in Table 3. Overall, the study of the biological potential of numerous natural plant phenolic compounds still remains a topic of significant interest among the scientific community [80]. Despite all of those advances, the available knowledge regarding the responsible phytochemicals for biological potential, their mechanisms of action, the establishment of therapeutic and prophylactic doses, and even the occurrence of biochemical inter-relations, is considerably scarce [80]. Finally, the observed variability in the polyphenol and flavonoid content has been attributed to the different pepper cultivars [81] and the maturity stage [82] because the stages from green to red indicate a decreased total phenolic content in peppers.

Notwithstanding the ongoing discussion surrounding the fluctuations in phytochemicals within *Capsicum* spp., researchers posit that the presence of phenolics and flavonoids is more pronounced in the green and immature stages of these species [83,84,85]. The literature indicates that pepper seed byproducts, including chopped pepper seeds [59], pepper seed core waste [86], seeds [87,88], pepper seed oil [58,89], pepper seed flour [21], and protein hydrolysates obtained from red pepper seeds [90], among others, possess potent antioxidant activity. Consequently, the implementation of a botanical classification scale for the purpose of differentiating between concentrations is proposed, with foods being classified as low (0.10–39.90 mg kg^−1^), moderate (40–99.90 mg kg^−1^) or high (> 100 mg kg^−1^). The proposed classification system is rooted in the work of Howard et al. [81] and Peterson and Dwyer [91]. Consequently, the red pepper seed extracts were categorized as exhibiting elevated flavonoid content.

### 4.3. Fatty Acid Profile of Pepper Seeds: Saturated and Unsaturated Fatty Acids

The pepper seed oil of *Capsicum annuum* L. is rich in unsaturated fatty acids, with linoleic acid (C18:2) representing the predominant fatty acid, accounting for between 67.80% and 77.90% of the total fatty acids (Table 4) [1,21,52,55,56,57,58,60,62]. According to Table 4, the remaining total fatty acids are oleic acid (C18:1; 4.6–14.6%), which is classified as unsaturated, and the saturated acids are primarily palmitic acid (C16:0; 10.60–14.40%) and stearic acid (C18:0; 2.40–4.10%) [1,21,52,55,56,57,58,60,62]. As Koncsek et al. [57] have demonstrated, the fatty acid composition of pepper seed oil remained consistent irrespective of cultivar or growing season. No significant differentiation in fatty acid profile was identified between the *Podravka* and *Slavonka* species and cultivars [16,21]. Matthäus et al. [92] reached a similar conclusion, indicating that the variation in linoleic acid (C18:2) between *Capsicum annuum* cultivars was minimal (69.50–74.70%), as was the variation in the other determined fatty acids, such as C16:0 (10.70–14.20%), C18:0 (2.50–4.10%) and C18:1 (8.90–12.50%). Accordingly, the most dominant fatty acid in cold-pressed paprika seed oil was linoleic acid, with a percentage of 69.60 ± 2.30% [93].

The beneficial effect is obtained with a daily intake of 10 g of linoleic acid, and the replacement of saturated fats with unsaturated fats in the diet contributes to the maintenance of normal blood cholesterol levels [monounsaturated fatty acid (MUFA) and polyunsaturated fatty acid (PUFA) both being unsaturated fats] [16]. In a study by Koncsek et al. [57], it was demonstrated that 10 g of pepper seed oil, when used as a salad oil, can provide 7.0–7.4 g of linoleic acid, which is equivalent to 70–74% of the suggested beneficial minimum daily intake.

### 4.4. Characterization of Volatile Organic Compounds of Pepper Seeds

The processing of pepper seed samples, including thermal treatments like roasting and biological methods such as fermentation, can introduce significant variability or even generate artifacts in the resultant aromatic profile. Volatile organic compounds (VOCs) in pepper seeds are critical to their aroma and flavour, which influence both their commercial and culinary value. The categorization of VOCs can be approached through the lens of their biosynthetic origins, which can be broadly classified into the following groups: fatty acid derivatives, amino acid derivatives, terpenoids, phenylpropanoids/benzenoids, and species- or genus-specific compounds not involved in the major classes [94,95]. Pepper volatile compounds that have been identified in studies are mainly aldehydes, esters, terpenes and alcohols [96,97]. These compounds vary significantly among cultivars and tissues, with higher concentrations typically observed in placental tissues. Despite the existence of several studies on pepper aroma substances, the majority of these studies concentrate on fresh and dried pepper fruit and chili pepper [96,97,98,99]. However, factors such as the maturity stage and processes such as roasting or fermenting chopped peppers have been demonstrated to influence the profiles of these substances [17,97,100], with the potential to enhance existing flavours or create new ones.

Forero et al. [97] identified 140 constituents as the steam volatile components of chile pepper (*Capsicum annuum* L. var. *glabriusculum*) at two ripening stages (green and red) using GC and GC–MS. Hexyl isopentanoate, hexyl 2-methylbutanoate, limonene, hexyl isohexanoate, (E)-2-hexenal, isopentyl isopentanoate and (Z)-3-hexenyl isopentanoate were found to be the major components. During fruit maturation, the majority of volatile compounds decreased [97]. In general, green chile peppers have higher amounts of esters, with their fruity odour notes, than red fruits [97]. Attending to the differences in the number of total volatiles, which is higher at the green stage in comparison with the mature stage, it can be concluded that the green stage is better in terms of its flavour than the red stage [97]. On the other hand, Chen et al. [17] have reported on the VOC profile of chopped pepper seeds, with aldehydes, esters, and alcohols as the predominant groups. A key finding was the presence of five common key VOCs across all three chopped pepper seed cultivars: 2-pentylfuran, methional, ethyl 3-methylbutanoate, dimethyl disulfide, and nonanal [17]. These compounds are considered significant contributors to the aroma due to their relative odour activity values (ROAV ≥ 1) [17]. However, the study also revealed remarkable variations in the aroma profiles among the three cultivars, these differences being likely attributed to factors such as the origin and cultivar of the raw peppers, different production seasons, and the fermentation methods employed during chopped pepper manufacturing [17]. Finally, the identified VOC profile in chopped pepper seeds showed similarities to that of pepper and chili sauce products, with the presence of compounds like 1-octen-3-ol, methyl salicylate, and ethyl acetate [17]. This suggests that chopped pepper seeds have the potential to be used as a functional food ingredient and a natural flavouring agent in the food industry to enhance flavour.

For instance, roasting can induce Maillard reactions and lipid oxidation, leading to the formation of novel volatile compounds that were not originally present in the raw seeds, potentially skewing the authentic aroma characterization [100,101,102]. Red pepper seeds were subjected to a roasting process, with constant agitation, for 6, 9, 10 and 12 min at a temperature of 210 °C [100]. The oils were subsequently extracted from the roasted red pepper seeds using an expeller [100]. Thirteen alkylpyrazines were identified in the roasted red pepper seed oils:  2-methylpyrazine, 2,5-dimethylpyrazine, 2,6-dimethylpyrazine, 2-ethylpyrazine, 2-ethyl-6-methylpyrazine, 2-ethyl-5-methylpyrazine, trimethylpyrazine, 2,6-diethylpyrazine, 2-ethyl-3,5-dimethylpyrazine, tetramethylpyrazine, 2,3-diethyl-5-methylpyrazine, 2-isobutyl-3-methylpyrazine, and 3,5-diethyl 2-methylpyrazine [100]. The pyrazine content exhibited a marked increase with an increase in roasting time, with levels of 2.63, 5.01, 8.48, and 13.10 mg of total pyrazine being recorded per 100 g of oil from red pepper seeds roasted for 6, 8, 10 and 12 min at 210 °C, respectively [100]. The 2,5-dimethylpyrazine content in the roasted red pepper seed oil appeared to be the component most responsible for the pleasant nutty aroma of the oils [100]. Furthermore, an increase in the oxidative stability of the oils was observed as the roasting time was increased [100]. Johnson et al. [101] have hypothesized that alkylpyrazines contribute to the roasted peanut flavour. That study proposed that alkylpyrazines, specifically 2,5-dimethylpyrazine, 2-methylpyrazine, trimethylpyrazine and 2,6-dimethylpyrazine, contribute to the roasted peanut flavour in red pepper seed oil [101]. These findings corroborate earlier studies that had previously identified these alkylpyrazines in roasted peanut volatiles [102].

On the other hand, fermentation processes, driven by microbial metabolism, can produce a distinct suite of volatile organic compounds (VOCs) that are characteristic of the fermentation process itself rather than the inherent seed aroma [17,97]. These processing-induced alterations can complicate the accurate identification and quantification of the native aroma compounds, thus limiting the direct correlation between the measured volatile profile and the intrinsic aroma of unprocessed pepper seeds [17]. Therefore, careful consideration of processing methods and appropriate controls are crucial to minimizing the introduction of artifacts and ensuring the reliable analysis of pepper seed aroma. This research is of paramount importance in comprehending the compounds responsible for the roasted flavour, and it may have significant ramifications for the food industry.

## 5. Sustainable Agrifood Applications: Integral Valorisation of Pepper Seeds

### 5.1. The Condimentary Use of Pepper Seeds in Food

Pepper seeds are a common byproduct of the processing of pepper fruits. Their nutritional and functional characteristics have been well documented, and they may be regarded as a potentially valuable raw material for the production of oil or the extraction of protein and fibre [21]. The extraction of oil from pepper seeds can be achieved through the utilization of a cold-pressing method, whereby the residual defatted pepper seed meal, otherwise known as press cake, undergoes additional processing to yield high-value protein and fibre [64]. An additional strategy for enhancing the viability of this byproduct could be to grind the pepper seeds after drying, thereby producing a flour that can be utilized in the creation of novel and functional products with more favourable chemical and sensory properties due to the incorporation of parts of pepper fruit [35,103]. As can be observed in the Table 5, some studies have focused on the valorisation of pepper seeds as byproducts to develop new products [82,104,105]. Bostanci et al. [64] studied the valorisation of capia pepper seeds, which is a waste product of capia pepper processing, in food applications, specifically in the development of innovative spreadable pastes. Two formulations, a chocolate type and a molasses type, were evaluated [64]. Both formulations included 23.7 and 30.1% pepper seed flour, chocolate liquor or molasses as aroma source, and sugar, palm oil, citric acid, potassium sorbate and lecithin as the ingredients [64]. The developed spreadable pastes were found to contain elevated levels of linoleic acid, sterols, tocopherols and dietary fibre, in comparison with commercially available products of a similar nature [64]. In the study conducted by Yilmaz et al. [104] the capia pepper seed flour possessed a characteristic peppery flavour. Nevertheless, these overall acceptable sensory properties have permitted the successful incorporation of pepper seed flour in quantities of up to 20% in the development of vegetable and spicy sauces [104]. Furthermore, Guo et al. [105] conducted a study on the development of new hot pot sauces based on pepper seed press cake. The results obtained from this study indicate that enriching the sauce with 5–10% of pepper seed press cake increases the content of palmitic and linoleic acids, enhances storage stability, and improves the rheological behaviour and textural properties [105].

Conversely, bioactive compounds extracted from red and yellow peppers have been employed as natural colourants in dairy products, such as yoghurt and isotonic drinks, enhancing their visual appeal, sensory attributes and nutritional value [35,106,107]. Furthermore, in the meat industry, *Capsicum* spp. extracts have been demonstrated to be efficacious in extending the shelf-life of beef and curtailing bacterial proliferation [108,109]. In a similar manner, the seeds of the pepper plant also contain bioactive compounds that could be utilised for these purposes, thus complementing and extending the established uses of the previously mentioned pepper extracts. A substantial number of studies have identified the potential of pepper seeds as a source of valuable compounds, including fibre and antioxidants, which could be used in the development of new products [64,104,105]. Pepper seeds contain a high concentration of fibre and antioxidants, and their grinding to produce flour could be a viable method of incorporation into various food products, such as bakery items and other processed foods, with the objective of enhancing their nutritional value. The oil obtained from the seeds, characterised by its richness in unsaturated fatty acids, has significant potential for use in the food industry. The oil’s high antioxidant content endows it with antimicrobial properties, rendering it an appealing ingredient for the preservation of foodstuffs. This approach may also provide a technologically feasible and economically sound solution to valorise food byproducts. However, there is currently a paucity of literature on the use of *Capsicum* spp. seeds as supplements, additives or coatings in food products. Nevertheless, given its nutritional value, the byproduct of *Capsicum* spp. seeds after a drying or roasting process could be another promising avenue for future agrifood applications and research.

The strategic utilisation of food industry byproducts is gaining significant traction, fuelled by a plethora of scientific studies demonstrating their capacity to enhance both the antioxidant and organoleptic qualities of food products. As demonstrated by Çalişkanlar et al. [110], the incorporation of pomegranate and grape seed powder into yogurt production has been shown to enhance the phenolic and antioxidant profiles, thereby positively impacting sensory attributes and increasing consumer appeal. An analogous development has been observed with pumpkin seeds, which have been incorporated into a diverse range of food products, including bakery items, dairy, meat, confectionery, and snacks, thereby significantly enriching their nutritional value with fatty acids, proteins, fibres, minerals, and bioactive compounds [111]. In addition, cocoa hulls have been explored as functional ingredients in biscuits, emphasising the versatility of these byproducts [112]. The potential of oils derived from passion fruit and grape seeds, recognised for their fatty acid content [113], further underscores this trend. Grape seed oil with its potent antioxidant and antimicrobial properties has been effectively incorporated into innovative food formulations such as yogurt, chocolate, canned fish, and sausages [114]. Regarding the pepper seed byproducts, sweet pepper seed oil can be used as a functional ingredient and natural preservative in beef burgers [115], and protein isolates from different types of pepper seeds have shown promise as food ingredients [90,93,116]. In the agri-food industry, defatted pepper seeds can enhance the nutritional profile of pasta [65] and improve the fatty acid composition of tarhana [117]. Furthermore, pepper seeds are a source of edible oil [56,77], and compounds such as allithiamine have been isolated for potential use as supplements [118].

Beyond oils and seeds, flours from mango seed and kernel have been used in porridge [119], while powders from wine industry waste have been used as oxidative stabilisers in perishable foods like chicken pâté [120], and grape pomace flour has been used to enrich bread [121]. The preceding studies indicate the considerable versatility of byproducts from the food industry, which have the capacity to enhance nutritional profiles and sensory attributes, while concurrently promoting sustainable food production. In this respect, pepper seeds are emerging as a comparable and sustainable alternative, with significant potential for byproduct valorisation through product development.

### 5.2. The Organic Pepper Seed Extracts as Plant Biostimulants

The use of elicitors or plant growth regulators (PGRs) at the preharvest stage is not a widespread practice among companies. This technology is still in the early stages of development and is currently the subject of experimental research. Elicitors that have been tested are based on a profile of different bioactive compounds. A notable example is the application of elicitors of salicylic acid (SA), jasmonic acid (JA), oxalic acid (OA), gamma-aminobutyric acid (GABA) and their derivatives, which have been used as tools to increase the yield and quality of various crops, including sweet pepper, artichoke, pomegranate and sweet cherry, lemon, plum, and table grape [122,123,124,125,126,127,128,129,130,131,132]. However, the main drawback of this approach is that obtaining the commercial compound places an economic burden on the company. Byproducts of fruit and vegetable processing include seeds, skin, pomace and peels, which are not usually consumed on a regular basis, but which contain bioactive compounds such as phytochemicals and secondary metabolites that are stored in the tissues [133,134].

It has been observed that these byproducts often have higher concentrations of bioactive compounds compared with the edible part of the fruit [135,136]. For example, pepper seeds have been shown to have a remarkable concentration of phenolic compounds, suggesting their potential use in the formulation of biostimulants for agricultural applications aimed at promoting growth and enhancing plant defence mechanisms. The biostimulant and bioprotective properties of exogenously applied extracts and phenolic compounds from aromatic plants have recently been evaluated [137,138,139,140,141]. Reported properties include improvements in seed germination, rooting, sprouting and fruiting, as well as antimicrobial, insecticidal, nematocidal and herbicidal properties [137,138,139,140,141].

As reported by Mejri et al. [142], novel resistance inducers have been identified from sugar beet byproducts that have the potential to improve resistance of wheat to *Zymoseptoria tritici*. In another study by Lujan et al. [143], phenolic extracts from pecan shells and hulls were shown to have the ability to reduce the infection of chilli plants by *Phytophthora capsici*. These findings provide a framework for further exploration of the use of secondary metabolite extracts as enhancers of plant defence responses against pathogens. However, there is a need to identify novel compounds that act as elicitors but which are derived from a plant byproduct, such as the byproduct seeds from green pepper processing. The biostimulant and bioprotective properties of exogenously applied extracts and phenolic compounds from aromatic plants have recently been evaluated. Reported properties include improvements in seed germination, rooting, sprouting and fruiting, as well as antimicrobial, insecticidal, nematocidal and herbicidal properties [137,138,139,140,141].

### 5.3. Nutraceutical Applications to Benefit Human Health

With the increasing prevalence of diet-related health concerns, the need for dietary supplements has become increasingly apparent. Pepper seeds are a source of essential compounds and oils, although their concentrations are low. Nutraceuticals are defined as compounds derived from foods or food components that provide health benefits by treating or preventing disease. They are characterised by the presence of antioxidants, dietary fibres, fatty acids and polyphenols [17,21,56,62]. The presence of bioactive compounds in fruit and vegetable wastes has been shown to serve as a reservoir to produce nutraceuticals [144]. Polyphenols are a heterogeneous group of bioactive compounds that play an important role in the nutraceutical industry. These compounds, which include phenolics, flavonoids, phenylparanoids, quinones, tannins and lignin, act as antioxidants and contribute to the prevention of chronic, degenerative and cardiovascular diseases [145]. The phenolic content of pepper seeds could be identified as a useful resource in preservatives, additives or dietary supplements (post-encapsulation). In addition, it has been postulated that the use and improvement of dietary fibre intake are bulk mediators responsible for improving faecal hydration, sugar absorption and intestinal motility [146]. These bulk mediators mainly include carbohydrates, hemicellulose, lignin, cellulose and pectin [147].

Seeds represent a significant proportion of plant byproducts and are a rich and valuable source of bioactive compounds of interest to the nutraceutical industry. Grape seeds, for example, are known for their high proanthocyanidin content, which has been used to improve cardiovascular health and reduce inflammation [148]. Pumpkin seeds, which are known for their high levels of phytosterols, have been shown to be beneficial for both prostate and cardiovascular health [149]. In addition, Kumar et al. [150] have documented that the high lycopene content of tomato seeds provides protection against oxidative damage and promotes cardiovascular health. Furthermore, cranberry seeds have been shown to have antibacterial properties due to the presence of type A proanthocyanidins [151]. On the other hand, chia seeds, which are high in fibre and omega-3, have been shown to improve digestive health and regulate blood sugar levels [152]. Finally, Mueed et al. [153] documented the beneficial effects of flaxseed on cardiovascular and digestive health. These effects are thought to be due to the alpha-linolenic acid (ALA), fibre and lignan content of the seeds.

As shown in the study by Viuda-Martos et al. [154], pomegranate seeds, which contain punicalagins and punicic acid, have been shown to have antioxidant and anti-inflammatory benefits. In addition, kiwifruit seeds, which are rich in fibre and antioxidants, have been shown to improve immune function and digestive health [155]. Given the growing interest in the use of byproduct seeds as nutraceuticals, pepper seeds are a promising option. Previous research has shown that *Capsicum* spp. seeds contain phenolic compounds and other phytochemicals with anti-inflammatory and antimicrobial properties, suggesting potential applications in digestive health and disease prevention. However, further specific research is needed to validate these findings. Nevertheless, the nutritional composition of *Capsicum* spp. seeds suggests that they could be added to the list of seeds used by the nutraceutical industry, following the trend of using plant byproducts for human health. Encapsulation techniques stabilise vitamins in red pepper seed oil [156]. In the context of healthcare and medicine, extracts from *Capsicum annuum* seeds have been shown to have antioxidant properties [157,158], while *Capsicum baccatum* seed extracts have been found to have anti-inflammatory properties [159]. Furthermore, capsicoside G, which is found in defatted pepper seeds, exhibits anti-obesity properties [160], while hot pepper seed extract has been shown to display antiproliferative activity against various tumour cell lines [161]. In this regard, the agricultural industry needs to invest in technologies that can convert byproducts and waste into bioactive compounds for optimal nutrient recovery.

### 5.4. Pepper Seeds as Suitable Ingredient for Nonfood Industries: Cosmetic and Biofuel Source

Considering the prevailing eco-friendly consumer and industrial trends, there is a significant interest in the exploration of bioactive compounds, raw plant materials, and plant extracts as natural ingredients or excipients for cosmetic or pharmaceutical applications. Vegetable oils are defined as organic compounds that are obtained through extraction processes from seeds and other parts of plants [162]. The chemical composition of these oils is dominated by triglycerides, diglycerides and fatty acids, including stearic, linoleic, oleic and linolenic acid, along with other minor constituents such as tocopherols and sterols. Fatty acids have been demonstrated to exert pivotal functions in the hydration, softness and suppleness of the skin, in addition to their contribution to the repair of the epidermis [163]. The properties of fatty acids have resulted in their frequent utilisation in the domains of cosmetics and dermatological pharmaceuticals.

It is evident that oils derived from seeds, fruits and vegetables have experienced a marked increase in popularity. This is primarily attributable to their distinctive characteristics and their favourable effects on the skin and hair. A prime example is argan oil, extracted from the seeds of the argan tree. This oil is highly prized for its moisturising and antioxidant properties and is an essential component in anti-ageing products [164]. In addition, rosehip oil, obtained from the seeds of the rose hip, is recognised for its ability to improve the appearance of scars and stretch marks, thanks to its high content of essential fatty acids [165]. A plethora of oils obtained from seeds and pits of fruits, including but not limited to pomegranate, fenugreek, poppy, blackcurrant, chokeberry, rosehip, perilla, elderberry and carrot, have been found to possess antioxidant properties. These oils provide a valuable complement to synthetic antioxidants, offering additional benefits to the skin [166]. The oils, when considered in conjunction with other compounds, offer a multitude of benefits, including but not limited to hydration, nourishment and protection against environmental damage. These components are widely regarded as being of paramount importance in the formulation of cosmetic products.

The byproduct of *Capsicum* spp. seeds has attracted increasing interest in the cosmetic industry, due to its composition, which is rich in bioactive compounds. Evidence suggests that the seeds contain oils with high levels of unsaturated fatty acids, including linoleic acid and oleic acid [1,21,52,55,56,57,58,60,62]. These acids are widely acknowledged for their emollient and moisturising properties. The incorporation of these oils into creams, lotions and body oils has the capacity to enhance skin hydration and restore its lipid barrier. In addition, *Capsicum* spp. seeds have been demonstrated to be a source of antioxidants, including carotenoids and polyphenols, which have been shown to protect the skin from damage caused by free radicals and oxidative stress. Furthermore, *Capsicum* spp. seeds have been found to contain phenolic compounds, including flavonoids, which possess both anti-inflammatory and antioxidant properties [15,21,56,77,78,79]. Consequently, these seeds could be utilised in the formulation of skin care products that aim to provide soothing and rejuvenating effects. In this regard, extracts obtained from these seeds could be incorporated into creams intended for use on the skin. The cosmetic industry has been engaged in an investigation into the potential of *Capsicum* spp. seeds as a natural and sustainable ingredient for the development of innovative and effective products, with the possibility of influencing the final product performance. Further study is required to ascertain the physical properties (colour, texture or permeability) and bioactivities (antimicrobial and antioxidant) of *Capsicum* spp. seeds, and their impact on cosmetic products.

Nevertheless, its application in cosmetics warrants consideration regarding safety, stability, and necessary analyses. In order to ascertain the suitability of the substance for topical application, it is imperative that comprehensive safety assessments are conducted. These assessments must include toxico-ecological studies and dermal irritation tests. Stability analyses, which evaluate oxidation rates and shelf-life under various storage conditions, are also essential to ensure product efficacy and longevity. Moreover, in order to identify and quantify any potential allergens or skin-sensitising compounds present in the oil, it would be necessary to carry out detailed chemical analyses that go beyond fatty acid profiling. As Cravotto et al. [167] have demonstrated, the fatty acid composition of pepper seed oil has yet to be fully elucidated. Further research is required to establish the safety profile and stability characteristics of this oil for cosmetic applications.

The bioaccessibility and bioavailability of bioactive compounds within pepper seeds are critical determinants of their efficacy in functional and nutraceutical applications. While specific data on pepper seed bioactives absorption are limited in the provided texts, pepper fruits are known to contain various compounds like carotenoids, phenolic acids, flavonoids, and vitamins C and E [168]. Capsaicinoids, though more associated with the fruit’s pungency, may also be present in seeds and possess antioxidant and anti-inflammatory properties [169]. For functional and nutraceutical uses, the bioaccessibility of these compounds—their release from the seed matrix during digestion—and their subsequent bioavailability—absorption and systemic circulation—are paramount. Factors such as the seed’s cell wall structure and the interaction of these compounds with the food matrix can influence these processes. Further research specifically investigating the bioaccessibility and bioavailability of pepper seed-derived bioactives is needed to fully elucidate their potential for functional food and nutraceutical development.

Processing methods, such as roasting and extraction techniques, can significantly influence the bioaccessibility of phenolic compounds from pepper seeds. Thermal processing, like roasting, may disrupt the seed matrix, potentially releasing bound phenolics and altering their chemical structure, which could either enhance or reduce their extractability and subsequent bioaccessibility during digestion [170]. Extraction methods employing different solvents and conditions can selectively isolate various phenolic fractions, thereby affecting the range and concentration of compounds presented for digestion. Studies using in vitro digestion models have been employed to simulate the gastrointestinal tract and assess the release of phenolics from the food matrix [171]. These models allow for the evaluation of how different processing techniques impact the liberation of phenolics during the oral, gastric, and intestinal phases. While the provided texts do not specifically detail such studies on pepper seeds, research on other plant matrices demonstrates that processing can lead to both an increased release of certain phenolics due to cell wall disruption and a decreased bioaccessibility of others due to degradation or polymerization [170]. Animal models could further elucidate the absorption, metabolism, and systemic availability (bioavailability) of these processed pepper seed phenolics, providing a more comprehensive understanding of their potential in functional and nutraceutical applications.

Regarding biofuels and biomass sources, the oil obtained from pepper seeds has emerged as a potentially viable source to produce biodiesel, owing to its abundant fatty acid composition, which is comparable to that of other vegetable oils commonly utilised in the biofuel industry. The transesterification process, which involves the conversion of oil into biodiesel, can be applied to this byproduct, resulting in the generation of a renewable and biodegradable fuel. The study carried out by Toghiani et al. [172] demonstrates the potential for the utilisation of pistachio byproducts in the production of fungal biomass, characterised by its high protein content, through the fermentation process with edible fungi. This finding could have significant applications in the field of animal feed and biorefinery. Moreover, a recent review has investigated various valorisation techniques to produce biofuels such as biodiesel, biogas, biohydrogen and fuel pellets from fruit residues and byproducts. A plethora of fruits has been documented as suitable for biofuel production, including mango, pineapple, banana, papaya, avocado and watermelon [173].

Pepper seeds have been found to contain a substantial oil content (11.00–23.65 g 100 g^−1^, up to 26.01% dry weight), thus positioning them as a potential resource for valorisation. The abundant fatty acid composition of pepper seeds has been shown to be comparable to that of other vegetable oils commonly utilised in the biofuel industry. This positions pepper seeds as a potentially viable source for producing biodiesel via processes such as transesterification. The yield of grape seed oil is subject to significant variation when extracted using different methods. Research by Ubaid and Sainia [174] found that the extraction of p-cymene yielded the highest percentage of grape seed oil (19.46%). Comparable ranges for grape seed oil content are also reported (11.6–19.6% and 10.92–14.52 g 100 g^−1^ seed) [174]. In contrast, Duan et al. [175] do not quantify the oil yield from pumpkin seeds directly, focusing instead on pumpkin seed oil bodies (PSOBs). In addition, pepper and grape seed oils exhibit a high proportion of unsaturated fatty acids, with linoleic acid (C18:2) being the dominant component (69.50–74.70% and ~68%, respectively) [167]. Furthermore, grape seed oils are distinguished by their elevated levels of PUFA, which range from 62 to 69%, and these levels are influenced by the extraction method [174]. Consequently, pepper seed oil is emphasised as a potentially viable source for biodiesel production, given that its fatty acid composition is comparable to that of conventional biofuel feedstocks, with transesterification being proposed for biodiesel conversion. While grape seed oil’s high linoleic acid content aligns with biofuel feedstocks, its primary applications are in food and cosmetics. The fluted pumpkin pod husk has been identified as a bio-based catalyst for biodiesel synthesis from neem seed oil. This finding indicates a valorisation pathway for pumpkin waste in the biofuel sector, distinct from using pumpkin seed oil as a direct feedstock.

In this sense, the utilisation of pepper seeds for biodiesel production has the potential to contribute to the principles of the circular bioeconomy. This is because it could reduce dependence on fossil fuels and minimise the generation of agricultural waste. The implementation of efficient technologies for oil extraction and biodiesel production from pepper seeds could stimulate the development of a more sustainable and diversified biofuel industry. In the domain of sustainable agriculture, pepper seeds have emerged as a promising resource, demonstrating remarkable versatility. These materials could be used as biomass for the purpose of thermal energy generation. The process under discussion involves the combustion of the seeds, either in a direct manner or after a densification process, such as pellet production. The resultant energy could be utilised for heating or electricity generation. This technology is particularly relevant in pepper-cultivating regions, as it facilitates the effective utilisation of agricultural waste. The utilisation of pepper seed biomass as a source of energy could have the potential to contribute to the reduction of greenhouse gas emissions and to the diversification of the energy matrix. Moreover, it has the potential to result in the establishment of a more sustainable economic model by assigning value to an agricultural byproduct that would otherwise be disposed of.

Quantifiable metrics demonstrate the impact of a circular bioeconomy: diverting approximately 85% of pepper seed waste from disposal, resulting in a measured reduction of 0.75 kg CO_2_ equivalent per litre of biodiesel produced compared with conventional diesel [176]. Energy efficiency analyses indicate a net energy gain of 1.8 MJ per litre of biodiesel produced via optimized extraction and transesterification processes [177]. A comprehensive life cycle assessment reveals a 30% decrease in overall environmental impact score, encompassing a 20% reduction in water consumption and a 15% decrease in land use intensity when compared with linear pepper seed management [178]. These figures underscore the potential of circular bioeconomy approaches to significantly enhance waste valorisation, mitigate carbon emissions, improve energy usage, and promote more sustainable practices in pepper seed processing and biofuel production.

Biodiesel production from pepper seeds demonstrates promising energy balance and reduced greenhouse gas emissions. By utilizing optimized solvent extraction, a substantial yield of crude oil can be obtained from pepper seed biomass [178]. The energy input for cultivation, harvesting, and processing is estimated at 15 GJ per hectare, while the energy output from the resulting biodiesel is approximately 55 GJ per hectare, yielding a net energy gain of 40 GJ per hectare [176]. Greenhouse gas emissions associated with the entire lifecycle, from seed to biodiesel combustion, are estimated at 0.5 kg CO_2_ equivalent per litre of biodiesel, representing a 60% reduction compared with fossil-based diesel’s 1.25 kg CO_2_ equivalent per litre [179,180]. These quantitative assessments suggest that pepper seed biodiesel offers a more energy-efficient and environmentally benign alternative to conventional fuels.

A comprehensive analysis is necessary to determine the economic viability of biodiesel production from pepper seeds, in comparison with established oilseed sources like soybean and rapeseed. The cost of pepper seed cultivation and harvesting, oil extraction efficiency and associated costs, which can vary significantly based on the method employed, and the transesterification process for biodiesel conversion are all to be considered. While pepper seeds have been shown to contain a significant amount of oil, a direct economic comparison would require detailed cost–benefit analyses. Such analyses would need to account for regional agricultural practices, potential yields per hectare, and the market value of pepper seed oil in comparison with other vegetable oils commonly used for biodiesel production. In addition, the potential for the valorisation of pepper seed byproducts following oil extraction could influence the overall economic feasibility of the process. In order to make a definitive assessment of its competitiveness, a thorough economic model incorporating these variables and comparing them against the well-established supply chains of soybean and rapeseed biodiesel would be essential in future studies.

## 6. Conclusions and Future Perspectives

In conclusion, pepper seeds, often discarded as waste, are a valuable byproduct with significant potential for application in sustainable applications. Their composition is characterized by a high content of oils, proteins and bioactive compounds, which can be extracted and utilized in various industrial contexts. For instance, pepper seed oil, with its favourable fatty acid profile, is suitable for use in the agrifood, pharmaceutical and cosmetic industries. This process has the dual benefits of reducing reliance on other vegetable oils and mitigating waste. Furthermore, the seeds contain high-quality proteins suitable for incorporation into animal feed or human food, thus contributing to food security and the diversification of protein sources. Furthermore, the seeds contain a high concentration of antioxidants and other compounds that have significant applications in the nutraceutical and cosmetic industries. The extraction of these valuable compounds allows the creation of highly value-added products from waste material, and the remaining seed material can be composted and reintegrated into the pepper crop cycle as organic fertilizer, thereby effectively closing the cycle and minimizing environmental impact. The integral valorisation of pepper seeds has been demonstrated to engender a reduction in waste, while creating new economic opportunities and contributing to the development of a circular bioeconomy.

Despite the recognised potential of pepper seeds as a valuable byproduct, there is a paucity of exhaustive scientific review literature specifically addressing their sustainable agrifood applications. There is a paucity of research in several key areas. For instance, the subject of pepper seed proteins has not been the focus of extensive study, and further investigation is required with regard to their diverse amino acid composition and variability among cultivars. Furthermore, there is a paucity of data concerning total phenolic and flavonoid contents, underscoring the necessity for more extensive quantification and characterisation across diverse cultivars and conditions. Moreover, despite advances in phytochemical identification, knowledge about the specific phytochemicals responsible for their biological potential, their mechanisms of action, and appropriate therapeutic or prophylactic doses is considerably scarce. With regard to the utilisation of *Capsicum* spp. seeds, there is a paucity of literature concerning their employment as supplements, additives, or coatings in food products. The potential for pepper seeds to act as a source of novel elicitors for plant biostimulant applications also requires dedicated investigation. While the composition of these samples suggests potential nutraceutical benefits, further research is required to validate these findings, emphasising the necessity for investment in bioactive compound recovery technologies. Finally, further research is necessary to ascertain the physical properties (e.g., colour, texture, permeability) and bioactivities (e.g., antimicrobial, antioxidant) of pepper seeds, and the precise impact of their incorporation into cosmetic products. Addressing these identified gaps is imperative to fully leverage the sustainable and economic potential of pepper seeds.

## Figures and Tables

**Table 1:** List of selected literature assessing the potential value of pepper seeds in relation to agri-food and non-food industry.

Type of Pepper Seeds	Outcome	Type of Industry	Scientific Gap	Reference
Pepper seed press-cake (byproduct of *Capsicum annuum* L. oil pressing)	Roasting enhances sauce quality, particularly at 140–170 °C, leading to decreased oil separation rate and improved storage stability.	Agri-food industry	Limited information regarding the application of pepper seed press-cake in food products.	[2]
Jalapeño pepper seeds and placenta (*Capsicum annuum* L.) derived from industrial processing byproduct.	The scalding process influences composition (e.g., decreasing capsaicinoids but increases total phenolics) and antioxidant capacity.	Agri-food industry/ non-food industry	A lack of comprehensive characterization of its bioactive compounds and antioxidant properties, particularly considering processing effects like scalding, limits its valorisation potential and efforts to reduce food waste.	[15]
Chopped pepper seeds (CPS) from *Capsicum annuum* L. A byproduct generated during fermented–chopped pepper production.	Nutritional richness: Rich in amino acids and fatty acids, predominantly unsaturated fatty acids and contains essential mineral elements. Flavour characteristics: 53 volatile organic compounds (VOCs) identified.	Agri-food industry	Lack of previous studies on the flavour compounds and nutritional composition of chopped pepper seeds (CPS).	[17]
Bell pepper (*Capsicum annuum* L.) seeds.	Rich in bioactive substances. Contain triterpenes, sterols, and fatty acids. Exhibit antioxidant potential. Show acetyl-cholinesterase-inhibitory properties.	Non-food industry	Further research is needed on immune-modulatory mechanisms and uses, and mechanisms regulating hypoglycaemic characteristics.	[20]
Dried pepper Seeds (*Capsicum annuum* L.), specifically the Croatian varieties Podravka and Slavonka.	Nutritional richness: Good source of protein, high content of dietary fibres. Seeds contain oil, rich in unsaturated fatty acids, primarily linoleic acid (C18:2). Bioactive compounds: Contain polyphenols and γ-tocopherol. Show antioxidant power. Oil quality: Pepper seed oil has a pleasant taste.	Agri-food industry/ Non-food industry	Further investigation is needed on product applications (culinary evaluations, added value product development) and the evaluation of nutritional, textural, and sensory traits influenced by fibre fortification.	[21]

**Table 2:** Nutritional profile and content of macronutrients, vitamins, minerals, and essential and non-essential amino acids of pepper seeds.

Macronutrients	Content by 100 g	References
Carbohydrate (g)	43.60–80.89	[17,55,62]
Dietary fibre (g)	26.00–61.00	[4,16,21,56,63]
Crude protein (g)	6.30–28.30	[5,17,21,52,55,56,60,62,63,64,65]
Crude fat (g)	11.00–23.65	[17,55,56,62,63,64,65]
Moisture (g)	4.48–5.96	[62,63]
Ash (g)	1.81–12.54	[17,62,63,65]
Vitamins	Content by 100 g	References
Vitamin A (IU)	3131.00	[66,67,68]
Vitamin C (mg)	127.70	[66,67,68,69,70]
Vitamin E (mg)	1.58	[66,67,68]
Vitamin K (Âµg)	4.90	[66,67,68]
Vitamin B3 (mg)	0.98	[66,67,68]
Vitamin B6 (mg)	0.29	[66,67,68]
Minerals	Content by 100 g	References
Sodium (mg)	2.35–2546.28	[17,56,62,63,65]
Potassium (mg)	306.89–921.33	[17,62,63,65]
Phosphorus (mg)	69.21–707.00	[17,62,65]
Magnesium (mg)	141.80–279.00	[17,63,65]
Calcium (mg)	38.80–174.71	[17,56,63,65]
Iron (mg)	3.01–17.49	[17,62,63]
Zinc (mg)	1.05–7.97	[17,62,63]
Manganese (mg)	0.38–4.05	[17,62,63]
Copper (mg)	0.72–3.33	[17,62]
Essential amino acids	Content by 100 g	References
Leucine (Leu; mg)	830–4006	[17,62,63]
Cysteine (Cys; mg)	413–3463	[17,62,63]
Histidine (His; mg)	192–1620	[17,62,63]
Lysine (Lys; mg)	537–1476	[17,62,63]
Phenylalanine (Phe; mg)	655–1344	[17,62,63]
Threonine (Thr; mg)	478–1188	[17,62,63]
Isoleucine (Ile; mg)	399–869	[17,62,63]
Tyrosine (Tyr; mg)	200–831	[17,62,63]
Methionine (Met; mg)	67–820	[17,62,63]
Valine (Val; mg)	85–760	[17,62,63]
Non-essential amino acids	Content by 100 g	References
Glutamic acid (Glu; mg)	1188–3668	[17,62,63]
Aspartic acid (Asp; mg)	1188–2030	[17,62,63]
Arginine (Arg; mg)	894–1731	[17,62,63]
Serine (Ser; mg)	558–1088	[17,62,63]
Glycine (Gly; mg)	617–894	[17,62,63]
Alanine (Ala; mg)	578–706	[17,62,63]
Proline (Pro; mg)	100–680	[17,62,63]

**Table 3:** Functional profile and content of phenolics and flavonoids of pepper seeds.

Fruit Type	TPC ^ϒ^	GA (ppm)	SC (ppm)	RA (ppm)	RV (ppm)	TFC ^Ϯ^	References
Jalapeño pepper seeds *(Capsicum annuum* L.)	10.01–13.09	-	-	-	-	-	[15]
*Podravka* pepper seeds (*Capsicum annuum* L.)	1.58	-	-	-	-	-	[21]
*Slavonka* pepper seeds (*Capsicum annuum* L.)	1.49	-	-	-	-	-	[21]
Red pepper seeds (*Capsicum annuum* L.)	21.50	-	-	-	-	0.04	[57]
Red pepper seeds (*Capsicum annuum* L.)	29.10	-	-	-	-	21.27	[77]
Red chilli seeds (*Capsicum frutescens* L.)	7.95–26.15	-	-	-	-	4.64–12.84	[78]
Red pepper seeds (*Capsicum annuum* L.)	22.30	5.53	14.52	23.87	0.00	-	[79]
Green pepper seeds (*Capsicum annuum* L.)	88.60	6.96	2.21	6.31	0.00	-	[79]

^ϒ^ TPC: Total phenolic content (mg GAE g^−1^ DW). ^Ϯ^ TFC: Total flavonoid content (mg QE, CAE or RU g^−1^ DW). Abbreviations: GA (gallic acid), SC (scopoletin), RA (rosmarinic acid) and RV (resveratrol).

**Table 4:** Fatty acid composition (%) of pepper seed oil (*Capsicum annuum* L.).

Fatty Acid	Content (%)	References
Palmitic acid (C16:0)	10.60–14.40	[1,21,52,55,56,57,58,60,62]
Stearic acid (C18:0)	2.40–4.10	[1,21,52,55,56,57,58,60,62]
Oleic acid (C18:1)	4.60–14.60	[1,21,52,55,56,57,58,60,62]
Linoleic acid (C18:2)	67.80–77.90	[1,21,52,55,56,57,58,60,62]

**Table 5:** Byproduct valorisation through product development.

Ingredient	Dosage (%)	New Product Development	References
Capia pepper seed flour	23.7–30.1	Novel spreadable pastes	[64]
Capia pepper seed flour	20.0	Novel breakfast sauce	[104]
Pepper seed press cake	5.0–10.0	Novel hot pot dipping sauce	[105]

## Data Availability

Not applicable.
